# “Throwing salt on wounds”: Covid-19 and a curriculum of embodiment

**DOI:** 10.1007/s11125-021-09561-x

**Published:** 2021-06-04

**Authors:** Zahra Kasamali

**Affiliations:** University of Alberta, 551 Education South, Edmonton, AB T6G 2R3 Canada

**Keywords:** Curriculum, Embodied knowledge, Connectivity, Rumi, Sacred ecology

## Abstract

The Covid-19 pandemic certainly amplifies the extent to which curriculum is adaptable, responsive, and proactive. These vulnerabilities, while daunting, can perhaps be welcomed as an invitation to reposition curricular priorities. Covid-19 reveals that an overreliance on the “curriculum as planned” and a continued absence of “the forgetful curriculum” will no longer suffice. The fragility of life and the sources that make life and living possible are often left out of curricular and policy imaginings. This article seeks guidance from Maulana Rumi’s story “The Graduate and the Boatman” and poem “One Task” to guide a possible reframing of a curriculum that remembers embodied knowledge and the ecological sources that unite all life forms. Embodied knowledge and ecological philosophies may offer ways to refocus curricula that can help youth to turn inward, courageously contemplate the difficult questions of life, and understand that unprecedented circumstances can be generative.

I re-entered the senior high classroom in winter 2020, with many hopes alongside the grade-10 English language arts and social studies students I was gifted to learn with and from. I was thrilled to begin the heavy lifting of a new term that found students enmeshed in transitioning from junior high and the stressors of balancing afterschool jobs, leadership club, 5:30 a.m. volleyball practice, band practice, cheer competitions, academics, and interpersonal relationships. In the midst of the multiple tasks and pressures that students were facing lurked extreme anxiety; fears of uncertainty; and the possibility of “not becoming somebody”, according to a 5-year plan. Despite these tensions, I invited my students to dwell within and contemplate that which makes them who they are. We explored conceptions of identity, belonging and joy through first engaging in a grounding activity wherein students were asked to draw their identities.

The request of identity drawings as a way to begin the new term unsurprisingly brought forth sheer horror and a sense of helplessness in many students. I watched students blankly glance at their empty sheets of paper. In the moments between this discomfort and distributing markers and pastels to the class, I received many questions about the assignment. Was the assignment for marks? Could students use their phones to look up ideas? Would drawing about hockey count as a representation of identity? As we proceeded together, many students commented that they found themselves perplexed. Why had locating themselves and openly sharing who they are among their peers become anxiety provoking and fraught with self-consciousness? How and why had self-silencing developed as a curricular and pedagogical normative experience?

Following the loathed identity drawing activity, I introduced all of my classes to Maulana Rumi’s powerful story “The Graduate and the Boatman”. Rumi was born in 1201 in Balkh, Afghanistan, during the Persian Empire. It was prophesized that his union with a divine friend (Shams of Tabriz) would deepen Rumi’s search for truth and connectivity (Dunn et al. 2010). The wisdom that flows from Rumi’s words inspires the courage to dwell within and to understand discomfort and obstacles as generative opportunities to grow and teaches the importance of living in balance and harmony with both living and non-living entities. In this story I shared with the class, Rumi wrote:

A certain conceited graduate traveled in a boat and spoke to the helmsman, asking him if he had ever studied languages.

The boatman replied that he had not, and the graduate told him that half his life had been wasted without such knowledge.

The helmsman was deeply hurt by this suggestion but at the time said nothing in reply.

Later, on the journey across the water, the boat was caught in a storm and the helmsman asked the graduate, “Tell me, do you know how to swim?”

“No”, cried the graduate in his pleasant tone. “Well graduate, the whole of your life has been wasted without this knowledge, for it is worth nothing in a sinking boat if you can’t swim”.

- Selflessness works where knowledge will fail,
- In surrender you’ll float, while in confidence sink,
- Die to the flesh, die to the mind,
- And the sea will carry you high. Bear your knowledge like a jug to the sea,
- Carry carafe to an ocean. (Dunn et al. 2010, p. 57)

I should note that I situate myself as a Pakistani-Canadian who was born and raised in a Sufic tradition of Islam. I was first exposed to Rumi’s poetry as a child, while in congregation with community members. Rumi’s poetry was shared to help us deepen our understanding of Allah (God) and all our relations. My parents also drew upon Rumi’s poetry and other mystical Muslim poets, including, Rumi’s beloved companion Shams of Tabriz and Rabia al-Basri. Rumi served as a guide and a reminder of what we can be if we commit ourselves to the acceptance of change and the possibility of transformation in our lives. His interpretations of Qur’anic verses, his teachings, and his relationship with sacred ecology in connection with Qur’anic teachings express the manifestation of differences as gifts of divine providence. For these reasons, it is important to remember Rumi’s connections to Islam, and the fact that he was raised in a Muslim family. Although Rumi contested the salience of organized religion and instead amplified the pervasiveness of spirit, his connections to Islam are far too often erased in Westernized accounts of his poetry and stories (Ali 2017).

After reading “The Graduate and the Boatman” as a class, my students participated in free writing and explored insights of the story that resonated with them. As we collectively unpacked the story, many students started to notice a particular theme emerge. They shared that formal knowledge, which they identified as “things you learn in school or curriculum”, is upheld as most important and the arbiter of all truth. We later discussed the danger of “sinking” into such knowledge alone. I invited students to consider what was left out of formalized knowledge. What might be at stake with an overreliance on this rendition of knowledge, which they so easily named as *curriculum*? The students further contemplated what the formalization of knowledge overrides and obscures. Musings surrounding intellectual vanity and pride dominated class discussions. However, there was still something absent from these reflections.

While we continued with our learnings, and weeks passed, the students continued to internalize aspects of Rumi’s “The Graduate and Boatman”, without consciously realizing their collective process. As we found ourselves approaching the first release of progress reports for the term, many students started to more openly address their anxiety as high school students. Our initial classes on approaching personal response writing brought us back to the very fears of our first days together approaching identity drawings. Many students begged and pleaded with me. They proclaimed in unison, “Please, no introspective writing!”, “We will write about anything else but ourselves!” Many expressed that self-reflection brought forth deep anxiety. They lamented that they did not know how to handle or how to address the anxiety that suddenly entered their lives post junior high. These conversations brought us back to the self. The students collectively identified that self-knowledge, attending to anxiety, learning who they are in deeper ways, and sharing gratitude were not part of their typical schooling experiences. The self was repeatedly left out of the curricular and pedagogical priorities of what it means to be a successful human being who would soon be entering the labor force. The absence of turning within, drawing upon inspiration from embodied knowledge and insights. accentuated their belief that life must be predetermined.

## “No, Covid-19 will not enter Canada”

The months preceding the first appearances of Covid-19 cases in Canada were met with cognitive dissonance. Covid-19 was described as an infectious disease that originated from afar and would evidently remain at a distance from the daily living of young Albertans. Class debates about the potentiality of Covid-19 entering Edmonton, Alberta, were initially passed off as fear mongering and a response to increased panic buying in the United States. While some students addressed their concerns that Covid-19 might become a pandemic, these thoughts were unpopular among my one hundred students. Once Covid-19 reached a pandemic status, it was as if salt was thrown on the wounds of already exposed anxieties, feelings of insecurity, uncertainty, and the imbalance that arises when one’s inner self is forgotten. The inability and willingness to address that which was unforeseen, unthinkable, and perhaps an impossibility for many amplified responses of denial and bewilderment to Covid-19’s presence and appearance on Albertan soil. What would become of an already emotionally exposed student body who continued to resist facing their inner selves? The fervent hand sanitizing and Chromebook cleaning in the days that followed, and gradual physical absence of students from the classroom, uncovered an already fragile educational system that continued to deny a needed balance between formalized and embodied knowledge. As schools across Canada gradually closed indefinitely for physical instruction, students found themselves entangled in a new reality. The closure of all that kept students busy and preoccupied, along with social distancing, summoned the generative possibility of self-facing and inner dwelling.

## What was left out and left behind? Alberta’s curricular imaginings of the ideal human being

I circled back to Alberta Education’s curricular foundations as I pondered pre-Covid-19 experiences alongside students. Alberta Education’s (2016) *Guiding Framework for the Design and Development of Kindergarten to Grade 12 Curricula* was a revision for curriculum design and architecture. It was written during Alberta’s New Democratic Party’s tenure, and the extent to which the United Conservative Party will honor its proposed commitments is unclear. The document positioned Alberta youth as “lifelong learners” who will be given the values to “explore and achieve” (p. 1). The document stated that “curriculum has a role in creating a positive future for Alberta, by preparing students for a more diversified economy” (p. 2). Focus on preparing for achievement of a particular kind and economic complexity was also reflected in the document’s overarching student vision: “Students are lifelong learners inspired to pursue their aspirations and interests; achieve fulfillment and success; and contribute to communities and the world” (p. 3). It is noteworthy that matters of success, achievement, and the ability to succeed on economic terms were frontloaded as having the greatest significance within Alberta Education’s visions for students’ success. While Alberta Education spoke to the importance of preparing students to respond to climate change, health matters, and Canada’s growing diversity, these matters were not situated as living in successful ways. It is evident that “statist interests” were privileged in the creation of the ideal human being Alberta Education had in mind.

Mcafee (2000) and Bartlett (2011) addressed what is meant by “statist interests” and its connections to economic pursuits. McAfee underscored that the connections between objectivity and solidarity originated in Athens in the 5th century (p. 166). Sophistry emerged out of attempts to receive monetary rewards in return for teaching students how to become successful citizens in the city. Central to Sophistry was its privileging of that which is tangible, immanent, and most visible. Sophists emphasized that truth could be found in what was visible, known in an immediate manner and transparent. McAfee underscored:

The sophists believed that young men could learn the virtues of excellences (arête) needed for citizenship by learning how to persuade and speak publicly, by learning as much as possible about their own culture and history—the literature and grammar of Greece past and present. (p. 166)

In this way, the pursuit of education is sought in accordance with the rule of the state (Bartlett 2011). Bartlett poignantly emphasized that “to know, in short is to be properly instructed in an interest in interest [whereby] one’s knowledge will translate into better wealth and one’s wealth into better knowledge” (p. 51). Subsequently, that which is worth knowing is that which serves self-interest and applicability to the state (den Heyer 2014, pp. 7–8). Such values are reflected in Alberta Education’s curricular documents, particularly in reference to preparing youth “to meet the opportunities and challenges of the future” (p. 1).

It is not overly assumptive to suggest that sophistic logics are embedded in the very makeup of formal education in Canada and can be understood as having deep resonance in Alberta’s programs of study. Donald (2009) explored the connections between “universal human insights and market logics following the rise of technological advancement in Europe and North America in the 19^th^ century” (p. 316). Desired economic growth was represented as a universal value, which had intimate connections to “codified presentations of knowledge” or programs of study that would help educators give students “the knowledge deemed most valuable” (p. 316). Donald underscored that “in accordance with market logics, education became associated with social Darwinism and curriculum as an expression of cultural capital and something of a gatekeeper to employability” (p. 316). Applying Donald’s illustration of the connection between curriculum and cultural capital, there is a connection to understanding Alberta Education’s curricular documents and programs of study as a “corporate and legal document” rooted in the interests of ‘expert groups’ that represent corporate bodies (den Heyer 2014, p. 12). Alberta Education (2016) defined its program of study, or provincial curricula, as “what students are expected to know, understand and be able to do in each subject and grade” (p. 1). At the same time, “teachers have the flexibility to determine ‘how’ students achieve the expected learning outcomes to bring the provincial curriculum to life in the classroom through meaningful learning activities” (p. 1).

Sophistry in connection to the promotion of statist interests helps to explicate the inextricable connections between the epistemic worthiness and perceived economic accumulation inherent in Alberta Education’s guiding philosophies, student visions, value systems, and curricular scaffolding. These approaches represent the continued philosophical currency of liberalism. The false universalism of liberal philosophy determines what is worthwhile for students to know. Alberta Education’s policy and curricular documents reveal the grooming of a human being that lives in ways that privilege “material recognition”. First, material recognition denotes how the majority of schooling and educational experiences are guided by ideologies, cultural assumptions, and practices that promote employability and success in the labor market alone. Second, material recognition reflects a totalizing philosophy for life that suggests that “becoming someone” in a worldly sense will leave individuals feeling satisfied with themselves, life, and living and will provide the necessary resources to attend to that which we cannot predict and control.

Material recognition’s focus on individualism and gratification ensures that the “acquisition of materials, feeling, and a sense of achievement becomes the common standards for individual pursuits” (Purpel and Mclaurin 2004, p. 53). Focusing efforts to attain what is external reinforces autonomous notions of being that override the connectivity of all life forms and distract from cultivating deeper relationships with oneself. While individualism in a liberal sense cautions against self-interest, such notions ought not to be conflated with deeper notions of connectivity, which necessitate a direct connection with oneself for life to go on. Purpel stated,

We have bought into a psychology that urges us to consider that we are responsible individually for our feelings and behavior and that we are responsible only for ourselves. While this may at one level enhance (properly) our own sense of personal responsibility, this attitude can and does serve to reduce the sense of our interdependence and our opportunities to help and support others. (p. 53)

Self-containment in this regard also promotes the fallacious notion that intellectual certainty and a predictable life course is guaranteed through abiding by prescriptive ways. Education that is anchored by self-containment promotes the cultivation of a human being that may devalue the “complex and elusive nature of truth and the vital importance of openness to and awareness of emerging consciousness” (p. 63). Conceptions of life, living, and survival that are primarily premised on the terms of *statist interests* and *material recognition* paradoxically contribute to disharmony with self, others, and nature in far-reaching ways.

These statist interests are brought to life through the foundations of the liberal Canadian nation-state, which centers on living and belonging on the terms of Canadian citizenship. Citizenship and citizenship education are frontloaded into Alberta curricular outcomes as essential to supporting a thriving Canadian economy that espouses the values of freedom, equity, pluralism, and responsible citizenship. The privileging of Canadian citizenship and citizenship education, as guided by liberal logics, flattens how other onto-epistemological traditions and sensibilities inform how responsible and ethical citizenship is enacted. Westheimer and Kahne (2004) posed the question “What kind of citizen do we need to support in an effective democratic society?” (p. 239). Westheimer and Kahne proposed that the categories of *personally responsible citizens*, *participatory citizens*, and *justice-oriented citizens* help to address their question. A personally responsible citizen is one who demonstrates responsibility within community contexts, pays taxes, obeys the laws, and recycles. A participatory citizen is an individual who is an active member of community organizations, organizes community efforts, and has an awareness of how government agencies work. A justice-oriented citizen is one who critically assesses social, political, and economic structures to see beyond surface causes and makes commitments to addressing social injustice (p. 240). The intent of these categories is to convey how individuals can come together in unified ways to support and uphold the nation and democratic citizenship. While I understand that citizenship education, as Westheimer and Kahne explicated, is meant to honor democratic values, the promotion of particular ideals to put into practice responsible citizenship ironically diminishes the equity that democratic nationhood promises. The promotion of prescriptive ways to honor citizenship is troubling in itself. This imposition curtails the possibility for individuals to connect with their inner beings as sources of guidance that inspire balance and connectivity with others within the context of the Canadian democratic nation-state.

While liberal philosophies have important offerings, I suggest that an overreliance on liberalism in curricular and pedagogical formulations is perpetuating imbalance among students. The false universalism of liberal philosophy continues to insist that there is a singular way to live wisely and well. Following Paine (1999), Turner (2006), and Venn (2002), I conceptualize the false universalism of liberal philosophy as the currency of liberal ideological structures in institutional spaces that normalize the rhetoric of sameness through difference. Conceptions of cosmopolitan communities within the Enlightenment period explicate the origins of sameness through difference. Venn (2002) contended “the idea that very different cultures across the world, indeed cultures that some thinkers at the time thought irreconcilable, could converge towards a cosmopolitan sameness that is inseparable from the twinned birth of European colonialism and of modernity” (p. 65). Creating a new order of cosmopolitan sameness was understood as necessary to alleviate the turmoil of encountering contrasting cosmologies, epistemologies, and ontologies. This construct was introduced and embedded in Euro-Western philosophies and presented as the norm for all cultures to mimic. Those who refused to emulate this model of life and living would be forever caught in a stagnant trap. This blueprint for life and living translated into the conceptual map for creating nation-states. For these reasons, modern nation-states, including Canada, follow the notion of *mission civilisatrice*, which affirms a commitment to the progress of the entirety of humanity. While liberal philosophies certainly offer a way to live and indicate that survival and balance is possible by maintaining fidelity to free market capitalism, a deepened sense of connectivity that can offer guidance regarding the “restoration of life to its original difficulty” is lost (Jardine 1992).

I venture to say that the false universalism of liberal philosophies contributes to shaping common sense approaches to curriculum and pedagogy. Following Britzman (2003), Kumashiro (2004), and Donald (2019), I conceptualize a curriculum of common sense as enhancing ideologies, which arise from the normalization and invisibilization of superficial knowledge and cultural assumptions. Britzman (2003) shared that superficial knowledge is “ensconced in the situations of visceral knowing [and] is made from the stuff of tacit understandings and practices” (p. 29) that organize the interpretation and enactment of educational life. Britzman emphasized that cultural myths also inform the production of superficial knowledge in educational settings. Cultural myths and their genesis as cultural assumptions inform ideological conceptions of the ideal citizen adults have in mind. In other words, adults imagine for their children what their futures ought to look like. These ideological conceptions assume that curricular goals must produce citizens who will eventually “hold social, political and economic power in a society” (Donald 2009, p. 106). Henry Giroux and Roger Simon (1984) contended that “[curriculum] represents an expression of struggle over what forms of political activity, orders of representation, forms of moral regulation, and visions of the past and future should be legitimized, passed on, and debated in specific pedagogical sites” (as cited in Britzman 2003, p. 56). The point to highlight here is that the process of legitimizing the curricular worthiness of particular ideologies is often given little attention. In this regard, curriculum is formulated on the basis of “normative worldviews” (Donald 2019, p. 106) and thus “become[s] a part of common sense” (Kumashiro 2004, p. xix). The normative worldviews espoused in common sense curriculum affirm how adults imagine their children as “becoming somebody” and receiving the benefits promised by liberal market capitalism.

While a “curriculum of common sense” (Britzman 2003; Donald 2019; Kumashiro 2004) positions Covid-19 as bringing to light vulnerability after vulnerability, the pandemic offers a significant pedagogical opportunity to think, act, and live otherwise. I am reminded of the loss of self and collective belonging students voiced following our transition to online instruction. While many students initially welcomed online instruction, they soon witnessed a palpable yet unexplainable void in their beings. The absence of meaningful connection to self and of building relationships with peers and friends that were not beholden to handheld devices and social media, heightened the isolation of Covid-19. Students who otherwise projected strong relationships in the classroom, out on the soccer field, and during swim meets were somehow unable to maintain these relationships as the loneliness of lockdown intensified. Online class time, office hours, and personal response writing uncovered a taste for sleeping in, learning from the comfort of one’s bed, and the initial excitement of a reduced timetable. Many students lamented their difficulties getting out of bed in the morning and could not make sense of these emotions. I attribute this absence of understanding and bewilderment to an educational system that continues to reinforce the curriculum as planned. “The curriculum as planned” (Aoki 1991) refers to the strict replication of curricular objectives and outcomes, as expressed in provincially mandated curricula, and contrasts with the organic enactment of a “curriculum as lived”, which opens up deep wonders, curiosities, and desires to ponder complexity. The curriculum as planned, in its prescriptive glory, insists that the answers to life’s deepest questions can only be attained externally and in objectified ways. This default approach to curriculum abandons our sacred ecological roots and undermines the fact that all beings are simultaneously related but different (Morris 2002). The unravelling of a neo-liberal curriculum during Covid-19, which leaves youth ill-prepared to attend to ambiguity, loss, and despair, welcomes rebuilding an ecological consciousness that deeply remembers our shared existence, enlivens the truths of inner knowing, and remains fiercely committed to the ebb and flow of life. I revisit Paramahamsa Yogananda’s (2020) words as I internalize these insights. Yogananda said,

Millions of people never analyze themselves. Mentally they are mechanical products of the factory of their environment, preoccupied with breakfast, lunch, and dinner, working and sleeping, going here and there to be entertained. They don’t know what or why they are seeking, nor why they never realize complete happiness and lasting satisfaction. By evading self-analysis, people go on being robots, conditioned by their environment. True self-analysis is the greatest art of progress. (para. 1)

Yogananda’s wisdom voiced how the deficiency of self-analysis and removal from one’s inner knowledge instills the notion that access to embodied knowledge and learning from them is of no consequence to life’s actualization. The dominance of “normative worldviews” (Donald 2019) furthered the fallacious notion that balance and living the good life can be attained through silencing uneasiness and tribulations. Rendon (2000) articulated that “we cannot understand the external world if we do not understand ourselves” (p. 9). A curriculum that dares to ask, “How is your heart, my friend?” may facilitate the recovery of connectivity and groundedness that is needed to teach individuals how to embrace suffering and reconceptualize difficulty as a necessary source to collective renewal and survival. Notably, various Eastern cultures from Arabic, Farsi, and Urdu linguistic traditions do not ask the question “How are you?” Rather, questions inquiring into another’s well-being are literally translated as “How is your heart?” and speak to one’s inner state in the present moment (Safi 2020).

Much of Rumi’s poetry and stories encourage attentiveness to the origins of worry, uncertainty, and wounds as essential to healing, growth, and learning. His words beckon a return to complexity and serve as a reminder that unearthing the deeper questions of life is in fact possible. Rumi’s poem “One Task” (*Fihi ma Fihi*: Discourse 4) examines what is taken into consideration to uphold life and living on a daily basis:

- There is one thing in the world that you must never forget.
- You may forget everything else except that one thing, without any cause for worry.
- However, if you remember and take care of everything else but forget that one thing, you will have accomplished nothing.
- It is as though a king were to send you to a village on a specific mission.
- You go and perform a hundred other tasks, but if you neglect to take care of the task for which you were sent, it is as though you did absolutely nothing.
- The human being has come into the world for a particular purpose.
- If he does not accomplish that purpose, he will have done nothing at all.
- We offered the trust to the heavens, and the earth, and the mountains: and they refused to undertake it, and were afraid of it, but the human being undertook it—but, surely, he has been unjust to himself, and foolish (Helminski and Helminski 2012, p. 24).

I interpret Rumi’s words as directly expounding the Qur’anic teaching of *Ashraf al-Makhluqat*, which is understood as a sacred gift from the Creator that entrusts human beings with the lofty responsibility of ensuring that creation remains protected; is well taken care of, and most importantly, maintains balance and harmony for all life forms. This responsibility is imbued in humility and meaning making, which can be espoused through learning from the signs embedded in our relations; these signs inspire communication among our relations, renew life, and actively promote and sustain connectivity. Shainool Jiwa (2020) expressed this thought and shared that “One of the key manifestations of God’s *rahma* (mercy) is His communication with humanity. Divine communication occurs through His numerous *ayat* (signs [i.e., of the Creator’s presence in relation to Qur’anic philosophies]) in the creation and through His Prophets” (para. 8). This rahma, through the appearance of difference as it arises from ayat*,* is a deep source of guidance but also a task that requires careful attention. Jiwa (2020) further underscored that, from Qur’anic sensibilities, human beings are gifted as the most noble of creation because they are appointed as Allah’s *khalifat*, or vicegerents on earth. This designation positions human beings as the “caretakers of creation and [as] accountable for its well-being to their Creator, who is the Sustainer of all worlds” (para. 9).

Rumi subsequently refers to this Qur’anic cosmological teaching as the “One Task” that ought not to be forgotten. Rumi’s emphasis that the “human being has come into the world for a particular purpose. If he does not accomplish that purpose, he will have done nothing at all” highlights how striving toward deeper connectivity is lost if cosmological guidance is ignored in the face of other tasks. Rumi’s referencing of the Qur’anic ayat that “We offered the trust to the heavens, and the earth, and the mountains…” addressed the gifting of the khalifat. Rumi elucidates the Qur’anic teaching that our more-than-human relatives were initially gifted with the responsibility of khalifat but did not accept this task for fear of being unable to uphold this promise. Rumi cautions that while human beings accepted the khalifat, they have not honored this position in ways that are required to support such a lofty responsibility. Thus, Rumi emboldens our human tendency to forget that our life and living are in fact beholden to other entities. He compels us to reflect upon the potential results for forgetting this “One Task”.

Rumi’s words emphasize that “there is one thing in the world” that must never be forgotten or forsaken. He further expresses that human beings have undermined the trust that was promised to the heavens, earth, and mountains. His insistent words directly refer to sacred ecology, or the entities that bestow life. Forgetting the providence of the sun, wind, and water and the creative potential of sacred ecology is likened to forgetting the meaning of life. Rumi cautions that a failure to uphold the “One Task” of human beings is as though life has not been lived with purpose.

Remembering our shared ecological roots is integral to reconnecting and learning from the wisdom that flows from non-human entities, other human beings, and emotional and spiritual foundations of knowledge. Guidance from sacred ecology encourages the internalizing of embodied knowledge, or the interweaving of mental, emotional, spiritual, and physical learnings. Note that I refrain from defining “embodied knowledge” in definitive ways, due to the time it has taken for me to articulate understandings of embodiment in connection to Sufic wisdom and learnings from students over the years. Locating research that specifically addressed my emerging experiences and understandings of embodiment was rather challenging and contradicts the spirit and intent of this article.

Mark Johnson’s (1989) work on embodied knowledge underscored the importance of how “interactions, social encounters, and communicative exchanges that are critical for our survival and the enhancement of the quality of our experience” (p. 368) are interpreted and understood, because they are directly guided by our bodies and movements. These interpretations certainly offer important contributions regarding the role of our bodies with regard to how we carry and interpret experiences. However, this particular interpretation does not directly speak to the ways in which the specific parts of our being guide connections to intuitive insights. For these reasons, I refer to Rumi’s examples of an individual who persevered to recover their inner voice throughout their life as sources of inspiration that inform what embodied understanding refers to in the context of this article. My conceptions of embodied understandings are specifically connected to recovering from our “ecological amnesia” (D. Donald, personal communication, November 12, 2019) and living in ways that uphold the “One Task”.

Rumi conceptualized ecological principles based on this ethic and explained that nature guides and instructs us to refrain from the compartmentalization that occurs when being is solely intellectualized (Clarke 2003). Ecology, in its different manifestations and presentation of opposites (e.g., the transition of night to day, growth from the earth, and the metamorphosis of a caterpillar to a butterfly), indicates the interplay between similar and different life forms. All life forms are granted with unique knowledge that offers guidance if individuals are willing to pay attention to these signs of life. Ecological philosophies in this regard, through the coexistence of different life forms, teach the importance of the creative potential of interpersonal relationships and the strength they offer (Morris 2002). The wisdom that flows from sacred ecology offers ways to reimagine thinking, learning, and relationships. Morris stated:

A failure to be in touch with the earth means that we, as human beings, have to move out of our way, move out of our heads and stop thinking about thoughts as an isolated event. And we have to stop thinking about ourselves as isolated creatures. (p. 581)

Morris’s words encourage a recovery from ecological amnesia. Such a recovery requires refraining from living in self-enclosed ways. Reconnecting with sacred ecology and the wisdom that flows from living and non-living entities may inspire the resilience and commitment needed to embrace individual and collective suffering. These cosmological and ontological notions are unfortunately overlooked under the prescriptive discourse on curricular and educational landscapes, as guided by liberal ideologies. Maxine Greene (1993) voiced how the prescriptive nature of diversity orientations impose “a single standard of humanness” (p. 212). As a way to recover what is lost from the life-taking capacity of such impositions, Greene advocated for honoring human beings “in terms of open possibility” (p. 213). Greene located art as a source of awakening that can “guide other possibilities of existing, of being human, of relating to others, of being other” (p. 214) that beg centrality in curriculum and pedagogy. Drawing upon Greene’s insights on difference and diversity, I suggest that connecting with sacred ecology can also inspire other possibilities of living and relating that do not begrudge complexity and obscure suffering.

Exploring these wonderings necessitates revisiting “our focus on education as an epistemological rather than an ontological question” (den Heyer 2018, p 10). Kent den Heyer pondered the ways in which educational focus can shift from an epistemological focus to who we are as humans ontologically speaking. He further shared:

Is there a human nature, for example? What capacities are all humans potentially capable of expressing? What is our relationship to the nonhuman world, from the menstrual moon to innumerable microbes in our guts? Ontologically, what is and therefore should be our relationship to the earth—the very humus of our being? What of our schooled disciplines can help us all address such ontological questions? (p. 10)

These notions speak to conceptions of educating for identity that is informed by sacred ecological insights. Morris (2002) stated,

Identities are constructed around reflections and relations; identities are invented and imagined around others and the world. Ecological consciousness co-constructs identities with others (human and non-human). In fact, the animals and plants are not absolutely exterior to us, but are part of us. (p. 583)

Morris’s curricular and pedagogical framing of ecological consciousness articulates a way to experience a deepened sense of connectivity between self and other life forms. Learning from the wisdom of sacred ecological teachings on curricular and pedagogical landscapes may honor how all life forms are wired for relationships that promote the nourishment of self and others. The etymology of consciousness explicates the term as “internal awareness” and the “state of being aware of what passes in one’s own mind” (Online Etymology Dictionary 2019).

The absence of purposefully cultivating inner awareness in curricular and pedagogical formulations is concerning because it closes off the generative potential for learning from embodied knowledge, kinship with human beings and our more-than-human relatives, varied lived experiences, and different ways of knowing and being. The genesis of an anthropocentric curriculum impedes possibilities to learn from transformative sources of meaning making and renewal. Curricular philosophies and policies that continue to forget these relationships may inadvertently encourage further imbalance and the fear of the unknown among youth. Knowledge and meaning that flow from other life forms may support nourishment and the continuity of life and greater trust in embodiment to encounter the unprecedented.

## Toward a curriculum of embodiment

Gadamer (1975) proposed that “understanding begins when something addresses us” (p. 249). A continued allegiance to normative worldviews, via the curriculum as planned (Aoki 1991) reinforces anthropocentric conceptions of life and living. These ideologies interfere with the potential for students and educators to reconnect with the gifts of sacred ecology and embodied knowledge. Covid-19, within the situation of education and schooling, further exposes that current curricular outcomes are unable to support “confront[ing] the suffering of our own selves let alone the suffering of others” (Safi 2020). Perhaps a curriculum as lived (Aoki 1991) that intermingles with ecological philosophies may awaken a return to refocusing and trusting embodied knowledge as imperative and generative sources of inspiration that guide curricular underpinnings and pedagogical priorities in K–12 classrooms. Such a curricular repositioning may then reconceptualize how might we attend to the isolation we are feeling during Covid by turning inwards?” (Safi 2020).

As I ponder forward, I am reminded of the unending difficult decisions colleagues and I have had to continue to make regarding the education of youth. These painful choices have at times involved reducing opportunities to help anchor youth toward learnings that are not dependent on the formal structure of schooling, curricular outcomes, and the pressures of preparing to successfully complete diploma examinations. In Alberta, Canada, diploma examinations for grade-12 courses have three purposes: “certify[ing] the level of individual student achievement in selected grade 12 courses, ensur[ing] that province-wide standards of achievement are maintained and report[ing] individual and group results” (Government of Alberta 2020, para. 2).

Paradoxically, the Covid-19 pandemic has offered the space to thoughtfully engage in learnings that illuminate that which is occurring here and now and the pedagogical nature of process. This focus has shifted to lingering in the moments of day-to-day life and the balance that such dwelling beckons. The modification to online delivery has been central to this process. I have spent much time speaking with students on the phone on a weekly basis or connecting via videoconference. While these conversations were approached with the sole purpose of receiving additional support for assignments, they have morphed into beautiful opportunities to share. Such truth sharing has opened up daily experiences of living in a pandemic context, balancing family priorities, helping younger siblings with their online learning, attending to the health of ill family members, learning to better emotionally attend to these realities, reminiscing on balancing the return to work, enjoying the outdoors, creating sidewalk art, and partaking in daily meals with family members.

I am thankful for the wisdom that flows from these moments of more present communication that welcomes deeper connectivity. The busyness of completing curricular outcomes and all that encompasses a full school day in the lives of adolescents and has come to be associated with a sense of self-worth (Safi 2020) has necessitated slowing down and refocusing on what is important. The flux between micro and macro responses to Covid-19 has opened up the inner beings of many students. This shift is reflected in a visible change in the quality of expression among students, who are embracing their vulnerabilities and writing from their core. Many students who initially resisted personal response writing and reflective practice are engaging with greater courage and an openness to that which cannot be predicted.

Connecting short stories and film to varied lived experiences that reflect the fragility of life is creating a curricular ethic that does not forsake embodiment, the mundane, and emotional knowledge. What was deemed most worthy in curricular and pedagogical sites is expanding to a deeper consciousness that does not take for granted the gift of a new day, the creative energy that flows from our relationships, and learning how to collectively respond to grief. Slowing down from external affairs in response to Covid-19 has inadvertently generated the ideal conditions needed to “find composure in the face of what we have encountered” (Jardine 1992, p. 3). The inevitable presence of the unprecedented need not be interpreted as foreclosing life and living. Rather, “throwing salt on wounds” can be reimagined as a necessary invitation that emboldens both the visible and unseen parts of ourselves (Safi 2020) and places unwavering trust in wounds as a facet of light that renews life; “our lives are already interwoven and interconnected” (Safi 2018, pp. 45–47).
